# Prediction of Smoking Habits From Class-Imbalanced Saliva Microbiome Data Using Data Augmentation and Machine Learning

**DOI:** 10.3389/fmicb.2022.886201

**Published:** 2022-07-19

**Authors:** Celia Díez López, Diego Montiel González, Athina Vidaki, Manfred Kayser

**Affiliations:** Department of Genetic Identification, Erasmus MC University Medical Center Rotterdam, Rotterdam, Netherlands

**Keywords:** human microbiome, trait prediction, smoking status, prediction modeling, class imbalance, data augmentation, machine learning, saliva microbiome

## Abstract

Human microbiome research is moving from characterization and association studies to translational applications in medical research, clinical diagnostics, and others. One of these applications is the prediction of human traits, where machine learning (ML) methods are often employed, but face practical challenges. Class imbalance in available microbiome data is one of the major problems, which, if unaccounted for, leads to spurious prediction accuracies and limits the classifier's generalization. Here, we investigated the predictability of smoking habits from class-imbalanced saliva microbiome data by combining data augmentation techniques to account for class imbalance with ML methods for prediction. We collected publicly available saliva 16S rRNA gene sequencing data and smoking habit metadata demonstrating a serious class imbalance problem, i.e., 175 current vs. 1,070 non-current smokers. Three data augmentation techniques (synthetic minority over-sampling technique, adaptive synthetic, and tree-based associative data augmentation) were applied together with seven ML methods: logistic regression, k-nearest neighbors, support vector machine with linear and radial kernels, decision trees, random forest, and extreme gradient boosting. K-fold nested cross-validation was used with the different augmented data types and baseline non-augmented data to validate the prediction outcome. Combining data augmentation with ML generally outperformed baseline methods in our dataset. The final prediction model combined tree-based associative data augmentation and support vector machine with linear kernel, and achieved a classification performance expressed as Matthews correlation coefficient of 0.36 and AUC of 0.81. Our method successfully addresses the problem of class imbalance in microbiome data for reliable prediction of smoking habits.

## Introduction

In recent years, human microbiome research has elucidated the importance of microbes in the host's wellbeing and their interplay with different phenotypes (Cho and Blaser, 2012; Gilbert et al., 2018). Human microbiome research is currently moving from characterization and association studies toward translational applications. These include diagnosis of metabolic diseases, such as type 2 diabetes (Duvallet et al., 2017; He et al., 2018; Reitmeier et al., 2020), chronic inflammation disorders (Duvallet et al., 2017; Zhou et al., 2018; Su et al., 2020), and cancer (Duvallet et al., 2017; Poore et al., 2020; Su et al., 2020; Zheng et al., 2020) among others, as well as the prediction of the likely outcomes in personalized interventions, such as therapeutic response (Ananthakrishnan et al., 2017; Zhou et al., 2018) and nutrition (Zeevi et al., 2015; Asnicar et al., 2021). In more specialized applications, such as forensics, novel uses of the human microbiome have been reported to help in reconstructing the crime scene (Díez López et al., 2019, 2020), estimating the post-mortem interval of corpses (PMI) (Belk et al., 2018), or identifying the potential perpetrator(s) of crime (Woerner et al., 2019; Yang et al., 2019a). This current trend is possible due to advances in high-throughput sequencing technologies and bioinformatics analysis methods, together with the large amount of microbiome data that has become available from public repositories. Often, machine learning (ML) methods are preferred for data analysis, with the random forest model standing out as the most often used method so far.

Despite the great promises of ML methods in microbiome research and their application in trait prediction (Reitmeier et al., 2020; Su et al., 2020; Zheng et al., 2020), they also face practical challenges. Heterogeneity in methods, such as nucleic acid isolation or target region of the marker gene, is often encountered in cumulative microbiome datasets and is an obstacle for cross-study applications due to introduced study-specific technical variation (Debelius et al., 2016). Avoiding the pooling of data from different studies can bypass the study-specific effect issue, though greatly reduces the statistical power with negative effects on the reliability of the outcome. Additionally, microbiome data commonly suffer from imbalanced sample distribution (Khan and Kelly, 2020; Poore et al., 2020; Anyaso-Samuel et al., 2021). Particularly in (binary) classification applications, it is commonly the case that one class is overrepresented (majority class) while the other is underrepresented (minority class). This class imbalance leads to spurious high classification accuracy favoring the majority class, while research often focuses on the minority class, and limits the classifier's generalization (Japkowicz and Stephen, 2002; Abd Elrahman and Abraham, 2013; Ali et al., 2013; Thabtah et al., 2020). Some microbiome studies have reported problems in their classifiers due to the class imbalance issue in their datasets. These problems include the different classification performances over different datasets (Wang and Liu, 2020), the inability to perform accurate classifications (Bokulich et al., 2022), or even the classification of every sample to the same class (LaPierre et al., 2019). Therefore, the class imbalance should be considered in the data analysis approach. However, collecting data from more samples is often not viable, and therefore many public datasets come with serious class imbalance problems. Thus, researchers must explore novel methods for solving the class imbalance at the data and/or algorithm level (Japkowicz and Stephen, 2002; Abd Elrahman and Abraham, 2013; Ali et al., 2013).

At the data level, synthetic sampling methods have been suggested for microbiome research (Knights et al., 2011), though studies applying them are scarce. With these methods, to balance the classes, new samples are synthesized *in silico* based on existing minority class samples and added to the training set, an approach referred to as data augmentation. For example, the synthetic minority over-sampling technique (SMOTE) (Chawla et al., 2002) is one of the most widely used methods to deal with the class imbalance problem in real-life applications, and has been employed in some microbiome studies (Brooks et al., 2018; Wingfield et al., 2018; Chen et al., 2020; Gomez-Alvarez and Revetta, 2020; Mehta et al., 2020). An alternative is the adaptive synthetic sampling approach for imbalanced learning (ADASYN) (He et al., 2008). More recently, the tree-based associative data augmentation (TADA) method (Sayyari et al., 2019) has been proposed as a microbiome-specific data augmentation method, since it takes into account the phylogenetic relationship between the microbial taxa, but has not been widely applied by the microbiome community as of yet.

In this study, we investigated the predictability of individuals' smoking habits from saliva using publicly available microbiome data that unavoidably are class-imbalanced. Smoking is prevalent in the general population; therefore, smoking prediction from human biological materials, such as saliva, is useful in epidemiology and public health research, can be relevant for medical interventions, and may be of interest to other applied fields, such as forensics. Typically, in epidemiology, public health, and medical studies, smoking habit phenotypes are collected *via* self-reported questionnaires, which, however, are known to be unreliable (Rebagliato, 2002). Alternatively, they are collected *via* laboratory tests, such as cotinine measurements, a metabolite of nicotine, in biological samples like the serum, saliva, or urine. However, cotinine levels are not always available, or collecting them is not always affordable in clinical settings, and smoking classification heavily depends on a suitable threshold. More recently, approaches based on host epigenetics have been introduced *via* the detection of smoking-associated DNA methylation signatures, but issues arise regarding tissue specificity of epigenetic models, as well as model accuracy and suitable laboratory test development, given the large number of epigenetic biomarkers required for accurate predictions (Maas et al., 2019). Hence, microbiome-based prediction of smoking habits from saliva may provide a suitable alternative solution.

Previous studies have established the association between some saliva microbes and the host's tobacco smoking habit (Kato et al., 2016; Takeshita et al., 2016; Wu et al., 2016; Rodriguez-Rabassa et al., 2018; Beghini et al., 2019; Sato et al., 2020). More specifically, these association studies found that the abundance of some bacteria, such as those from the *Proteobacteria* phylum, is decreased in the saliva of smokers, while that of other bacteria, such as from the *Actinobacteria* phylum, is increased. However, at the lower taxonomic levels, there are some discrepancies between studies and study-specific associations. Notably, the largest available studies suffer from class imbalance with a ratio of about 1:5 between the minority class of current smokers and the majority class of non-smokers (Takeshita et al., 2016; Wu et al., 2016). Such class imbalance in the available microbiome data causes a typical and serious problem that needs to be solved by developing and applying suitable data augmentation methods to avoid a negative impact on the final prediction outcome.

In the present study, we deal with class-imbalanced saliva microbiome data for the purpose of predicting individuals' smoking habits. Our strategy consists of (i) optimization and validation of different data augmentation techniques and ML methods using nested cross-validation, (ii) identifying the best-performing approach for predicting smoking habits by taking class imbalance in the underlying microbiome data into account, and (iii) applying the best-performing approach for prediction modeling of human smoking habits from saliva microbiome data despite the underlying class-imbalanced data. The data and the code used are made publicly available.

## Methods

### Datasets

Publicly available 16S rRNA gene amplicon sequencing data and associated metadata from two different studies were obtained from the European Bioinformatics Institute (EMBL-EBI). The first study (Wu et al., 2016) (referred to as dataset S1 in this study) included two cohorts: the American Cancer Society (ACS) Cancer Prevention Study II (CPS-II) Nutrition cohort (*N* = 543) (Wu et al., 2016) and the National Cancer Institute (NCI) Prostate, Lung, Colorectal, and Ovarian (PLCO) Cancer Screening Trial cohort (*N* = 661) (Wu et al., 2016). The second study (Beghini et al., 2019) (referred to as dataset S2 in this study) included a single cohort from the New York City Health and Nutrition Examination Survey (NYC HANES) (*N* = 297) (Beghini et al., 2019). Dataset S1 comprised 454 pyrosequencing data, whereas dataset S2 comprised Illumina MiSeq data. We discarded samples based on the following criteria: (i) samples lacking metadata information for age, sex, and/or ethnicity, (ii) samples from donors <15 years old based on microbial community differences between youth and adults (Burcham et al., 2020), (iii) duplicate samples from dataset S1 to avoid data redundancy, and (iv) samples from non-smokers with second-hand exposure and “alternative” smokers from the dataset S2. The selected characteristics of the two analyzed datasets are described in Table 1. The setup of the experimental studies is described in further detail in Supplementary Table 1.

**Table 1:** Characteristics of the two saliva microbiome datasets used in this study.

	**Dataset S1** **(*N* = 1,088)**	**Dataset S2** **(*N* = 157)**
**Smoking status, *N* (%)**		
Never smoker	473 (43.5)	39 (24.8)
Former smoker	519 (47.7)	39 (24.8)
Current smoker	96 (8.8)	79 (50.4)
**Sex, *N* (%)**		
Female	429 (39.4)	88 (56.1)
Male	659 (60.6)	69 (43.9)
**Age group, *N* (%)**		
20–29	–	20 (12.7)
30–39	–	31 (19.8)
40–49	–	40 (25.5)
50–59	147 (13.5)	29 (18.5)
60–69	505 (46.4)	21 (13.4)
70–79	377 (34.7)	9 (5.7)
80–89	59 (5.4)	6 (3.8)
≥90	–	1 (0.6)
**Ethnicity, *N* (%)**		
European	1,028 (94.5)	59 (37.6)
Non-European	60 (5.5)	98 (62.4)

### Processing of 16S rRNA Gene Amplicon Sequencing Data

The data from the two selected studies were processed separately. Primer sequences were obtained from the original studies and were removed from the raw sequencing reads using cutadapt (v.1.15) (Martin, 2011) by setting the minimum length to >100 bp. The resulting FASTQ files were quality-filtered and de-noised using DADA2 (v.1.12.1) (Callahan et al., 2016). We used recommended parameters that we only modified when needed for our own data. Briefly, in both studies, parameters maxNN and maxEE were set to 0 to avoid unambiguous nucleotides and “expected errors” in the sequencing reads, respectively. Additionally, in dataset S1 (single-end), parameter maxLen was set to 500, and in dataset S2 (paired-end), parameter truncLen was set to 200–150 based on the read quality profiles, making sure to maintain overlap between forward and reverse reads to merge them later. After sample inference of true sequence variants, an amplicon sequence variants (ASV) table was constructed for each study, and chimeric sequences were removed using the command *removeBimeraDenovo()* with default parameters. Subsequently, the naïve Bayesian classifier method was employed for taxonomy assignation using the expanded Human Oral Microbiome Database (eHOMD) (v.15.2) (Escapa et al., 2018) as reference. At this point, only high-coverage samples (>1,000 reads) were kept for downstream analysis, and species with mean relative abundance < 1E−04 across samples were discarded. Taxa counts were normalized using total-sum scaling (TSS) for relative abundance (Paulson et al., 2013) (dataset S1 vs. dataset S2; PERMANOVA Bray-Curtis *R*^2^ = 0.20, *q* < 0.05; PERMANOVA Jaccard *R*^2^ = 0.13, *q* < 0.05). Moreover, microbiome datasets are normally sparse and characterized by a zero-inflated distribution, where most taxa are not shared among the majority of the samples. This is magnified in cross-study applications with study-specific taxa, which can limit the generalizability of the applications. Based on this finding, we merged the two ASV tables from the two analyzed studies and filtered out study-specific taxa, keeping 124 species from 30 families that were common between the two datasets for downstream analyses (Supplementary Table 2) (dataset S1 vs. dataset S2; PERMANOVA Bray-Curtis *R*^2^ = 0.14, *q* < 0.05; PERMANOVA Jaccard *R*^2^ = 0.09, *q* < 0.05).

### Statistical Analyses

The overall differences in the saliva microbial communities between the smoking classes were calculated in QIIME 2 (v.2019.10) (Bolyen et al., 2019): current vs. never vs. former, and current vs. non-current (combined never and former). For this, the weighted UniFrac distance matrix was analyzed by analysis of similarities (ANOSIM) and permutation multivariate analysis of variance (PERMANOVA) where *q* values (*q* < 0.05 for significance) were obtained with default 999 permutations.

### Data Augmentation Techniques

For the prediction of an individual's current smoking habit (smoker vs. non-smoker), we aimed to employ a binary machine learning (ML) classifier. For that, data imbalance was a marked issue in our dataset with a ratio of about 1:6 between the minority class (*N* = 175 smokers) and the majority class (*N* = 1,070 non-smokers) (Table 1). The problem stems from the ML algorithms that assume an equal number of samples for each class, which would lead to spurious high classification accuracy, favoring the majority class and limiting the classifier's generalization. Therefore, we applied different data augmentation techniques to overcome the data imbalance issue at the data level in our dataset. We used two techniques commonly employed in different fields to handle data imbalance, namely, the synthetic minority over-sampling technique (SMOTE) (Chawla et al., 2002) and the adaptive synthetic sampling approach (ADASYN) (He et al., 2008), as well as a recently introduced technique specific for microbiome data named tree-based associative data augmentation (TADA) (Sayyari et al., 2019).

The general approaches to deal with data imbalance are over-sampling (increase the minority class), under-sampling (decrease the majority class), or a combination of the two. Particularly, SMOTE and ADASYN techniques differ in the generation of synthetic samples in the minority class (over-sampling). For that, SMOTE over-sampling pinpoints the samples belonging to the minority class in a Euclidean space, and a random sample is first chosen for which *k* of its nearest neighbors are found. A line is drawn between the original sample and one randomly chosen neighbor, where a new synthetic sample is generated at a random point along the line (linear combination of samples). The process is repeated generating the same number of synthetic samples for each original minority sample until a specific ratio between the minority and majority classes is reached or to equal the majority class. On the other hand, ADASYN adds random small values to the neighbor samples; hence, they are not linearly correlated to the original sample. By this, ADASYN considers a density distribution between the original sample and its neighborhood, which acts as the criterion to set the number of synthetic samples to be generated from each original sample. On another point, with the under-sampling approach, random majority class samples are dropped out until a specific ratio between the classes is reached. Both SMOTE and ADASYN techniques were implemented using the imbalanced-learn Python toolbox (v.0.6.1) (Lemaître et al., 2017) with default parameters. We employed a combination of over- and under-sampling methods, indicated as SMOTE-1 and ADASYN-1 in this study. In order to set the final ratio between the minority and majority classes, we used the following equation:


$$\begin{array}{l} t\;=\;|C_{min}-C_{max}|,\;over-sampling\;=\;\frac{\left|t-\;C_{min}\right|}{C_{max}},\\ \;under-sampling\;=\;\frac{C_{max}\;-\;t}{C_{min}} \end{array}$$

where *C*_*min*_ is the number of the minority class samples, *C*_*max*_ is the number of the majority class samples, and t is the absolute value of the difference between *C*_*min*_ and *C*_*max*_.

We also used the over-sampling approach alone, indicated as SMOTE-2 and ADASYN-2 in this study, by which the number of the samples in the minority class was equaled to the majority class.

The microbiome-specific TADA technique generates minority class synthetic samples based on a statistical generative model that takes into account the phylogenetic relationships between microbial taxa. We implemented TADA with default parameters, which equals the number of samples in the minority class with the majority class. For the rooted phylogenetic tree required as input, we used the merged ASV table of the two studies to obtain a single consensus sequence for all those sequences assigned to the same taxa at the species level. For that, we used the *ConsensusSequence* function in DECIPHER (v.2.12.0) (Wright, 2016) and subsequently performed multiple sequence alignment of the consensus sequences using MAFFT with auto parameter (v.7.310) (Katoh et al., 2002). A rooted phylogenetic tree was obtained using FastTree (v.2.1.11) (Price et al., 2010) with a generalized time-reversible (GTR) model.

### Machine Learning Methods

**Figure 1:**
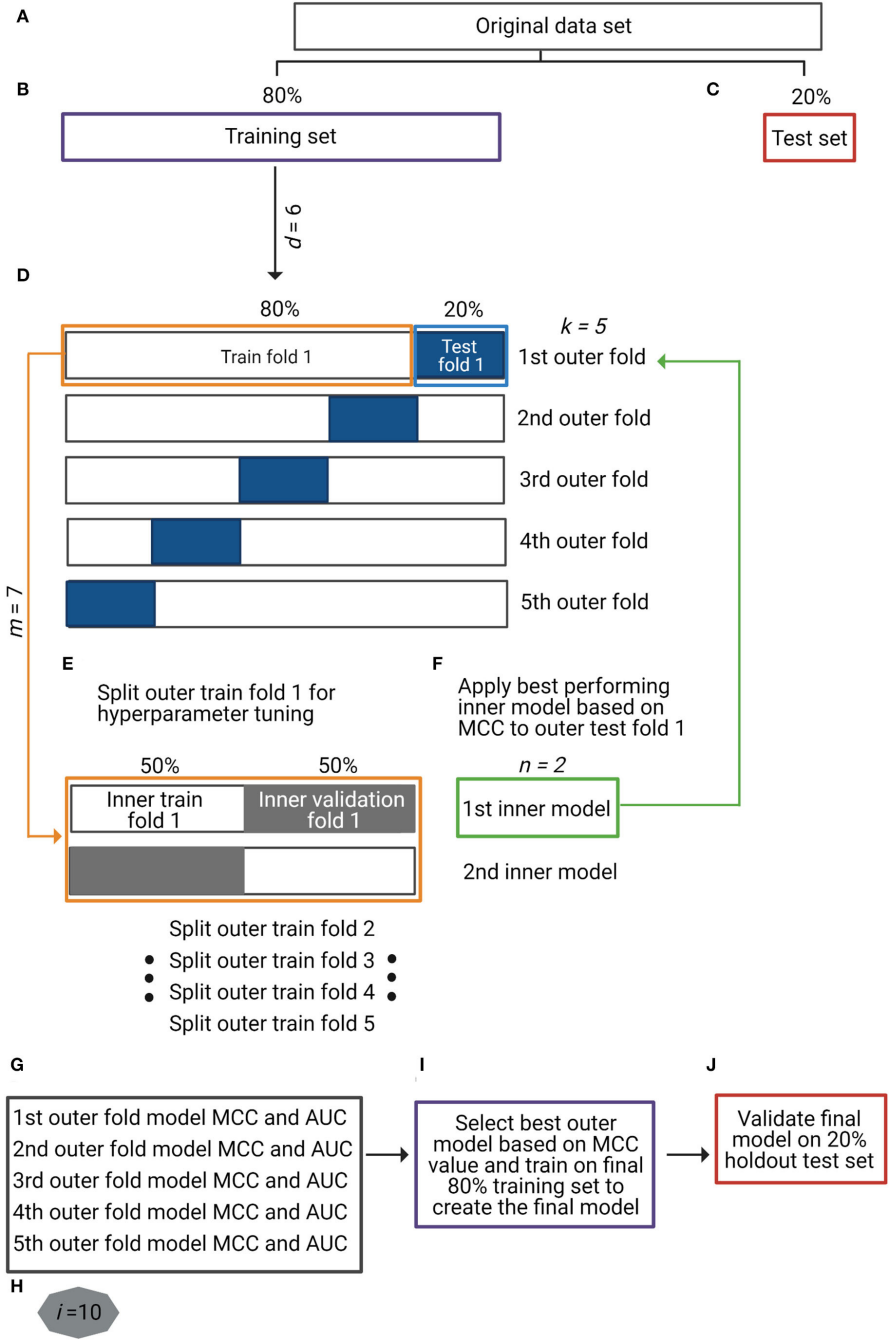
Overview of the study's analytical strategy. **(A–C)** The original dataset was split into a training set (80%) (purple box in **B**) and a holdout test set (20%) (red box in **C**) by maintaining the original ratio between classes in the partitions. Data augmentation techniques were applied to the training set, making a total of six different input data types (*d* = 6), including baseline non-augmented and differently augmented data types. **(D)** For the nested cross-validation (nCV) approach, the training set was split into five outer *k*-folds of training (80%) (orange box in **D**) and test (20%) (blue box in **D**) sets each. **(E)** Each outer *k*-fold was split into two inner *n*-folds of training (50%) and validation (50%) sets (orange box in **E**) in which seven different machine learning (ML) models (*m* = 7) were optimized and validated (inner models). **(F)** The best-performing *n*-fold inner model (green box in **F**) was applied to the corresponding *k*-fold test set (green arrow to blue box in **F**). **(G)** For each *k*-fold test set, two performance metrics were obtained: Matthews correlation coefficient (MCC) and area under the receiver operating characteristic curve (AUC). Repetition of steps **(D)** to **(G)** for all the input data types (*d* = 6) with ML method (*m* = 7) (total of 42 different approaches). **(H)** Repetition of steps **(A)** to **(G)** 10 times (*i* = 10) to control for introduced variation by data partitions. **(I)** Selection of the best-performing data type with ML method based on MCC metric and training on full final 80% training set to create the final prediction model. **(J)** Validation of final prediction model on final 20% holdout test set.

We evaluated seven different ML methods for binary classification (0: smoker, 1: non-smoker) included in the scikit-learn (v.0.23.2) Python package (Pedregosa et al., 2011): logistic regression (LR), k-nearest neighbors (KNN), support vector machine with linear (SVML) and radial (SVMR) kernels, decision trees (DT), random forest (RF), and extreme gradient boosting (XGBoost). LR is a parametric ML model that assumes a linear dependency between the input features (taxa) and the categorical outcome. The output of the logistic regression linear function is a probability *x* between 0 and 1, where if *x* < 0.5, the categorical outcome is 0 (smoker), otherwise it is 1 (non-smoker). KNN is a non-parametric model and as such supports non-linear solutions. It finds the Euclidean distances between a query sample and a *k* number of its closest samples (nearest neighbors) in the feature space and identifies their most frequent class label. SVM models take the data points and find a separating hyperplane between the two classes. SVML is a linear method that looks for linear dependencies among the input features to separate classes. SVMR is a non-linear method that adds an extra dimension to the data (kernel), so they become linearly separable and then project back the decision boundary to the original dimension using the dot product of two vectors in the feature space known as the kernel function. DT is a tree-based ML algorithm that mimics a decision diagram. Each input feature constitutes a node in the tree, where based upon a certain condition or rule splits into sub-nodes and extends until the leaf node that represents the classification decision (0 for smoker or 1 for non-smoker). Finally, RF and XGBoost are tree-based ensemble models that combine several models to improve their outcome predictions. RF generates a large number of decision trees on different subsamples and combines their outputs using averages at the end of the learning process. On the contrary, XGBoost combines the decision trees during the learning process for which it uses a gradient descent algorithm. By this, the mistakes done in a previous model are learned and improved in the subsequent model until no further improvement can be achieved. Hyperparameter optimization for all the ML models was performed using nested k-fold cross-validation (Figure 1E).

### Nested K-Fold Cross-Validation

Nested cross-validation (nCV) is a resampling procedure that enables both model optimization and evaluation (Krstajic et al., 2014). The difference between non-nested and nested CV approaches is that the former use the same cross-validation set for hyperparameter optimization and model evaluation, which biases the model to the dataset and leads to optimistically biased classifier's performance; in other words, non-nested approach leads to over-fitting in model selection. The nCV approach overcomes this by evaluating the ML algorithm and the model hyperparameters separately in multiple randomized partitions of the data (Cawley and Talbot, 2010), though it requires more computational time. In nCV, apart from splitting the original training set into *k-*folds of training and test sets (outer folds) (Figure 1D), each *k* training fold is at the same time split into *n-*folds of training and validation sets (inner folds) for model hyperparameter tuning (Figure 1E). The optimized model is then validated in the corresponding *k* test fold (Figure 1F). We employed a 5 × 2 (*k* × *n*) nCV where each of the first splits is named outer-fold (*k*) and each of the inside splits for hyperparameter tuning as inner-fold (*n*). Hyperparameter optimization for the seven ML models was performed using the *RandomizedSearchCV()* function in scikit-learn.

### Validation of Data Types With Machine Learning Methods

Since most ML algorithms operate trying to maximize the classification accuracy, spurious high classification occurs in imbalanced datasets by correctly classifying all or almost all the samples from the majority class at the cost of misclassifying many samples from the minority class. Hence, performance metrics, such as accuracy, or F1 score alone can lead to misleading results in imbalanced datasets (Chicco and Jurman, 2020). In contrast, the Matthews correlation coefficient (MCC) offers a balanced metric by considering the four confusion matrix categories: true positives (TP), true negatives (TN), false positives (FP), and false negatives (FN), according to the following equation:


$$\begin{array}{l} MCC\;=\;\frac{TP\times TN-FP\times FN}{\sqrt{\left(TP+FP\right)\times \left(TP+FN\right)\times \left(TN+FP\right)\times \left(TN+FN\right)}} \end{array}$$

For a high MCC score, the classifier has to correctly predict a high percentage of the samples in both the majority and the minority classes, independent of their ratios in the overall dataset, and hence is independent of data imbalance. MCC gives a score ranging from [−1 to +1], where 1 means perfect prediction, 0 random prediction, and −1 perfect inverse prediction. Based on this finding, in order to compare all the possible combinations of input data types, including original non-augmented data (Figure 1, *d* = 6) and the ML method (Figure 1, *m* = 7), we used the MCC metric. However, we also reported the AUC metric to show potential misleading results in those models with baseline non-augmented data (highly imbalanced). Comparisons among the different data types with a given ML method were performed in the R environment (v.3.6.1) using the Kruskal–Wallis and Wilcoxon tests. Significant *p-*values were determined with a cut-off value of 0.05 following Benjamini-Hochberg (BH) correction.

### Approach Setup

The original dataset (*N* = 175 smokers and *N* = 1,070 non-smokers) was split into a training set (80%) and a holdout test set (20%) maintaining the sample ratio between the classes (Figures 1A–C). Data augmentation techniques were applied to the training split: ADASYN-1 (over- and under-sampling), ADASYN-2 (over-sampling alone), SMOTE-1 (over- and under-sampling), SMOTE-2 (over-sampling alone), and TADA. We evaluated a total of six training data types (*d* = 6), including the original non-augmented and the five augmented data types (Figure 1B). Considering each data type separately, we optimized and evaluated seven ML methods (LR, KNN, SVML, SVMR, DT, RF, and XGBoost) (*m* = 7) using an nCV approach as explained before (Figures 1D–F). This entire process was repeated 10 times (*i* = 10) (Figure 1H), aiming to avoid introduced variation by the original data partitions. The performance metrics (MCC and AUC) resulted from the validation of each optimized model in the five outer test folds (*k)* over 10 times (*i* = 10) (total of 50 (5 ^*^ 10) resulting values for each metric) (Figure 1G). The best-performing data type with the ML method was based on the highest resulting MCC value (Figure 1I), and the final classifier trained in the final 80% training set (Figure 1I) was validated in the final 20% holdout test set (Figure 1J).

## Results

### Saliva Microbiome Data

The data comprised saliva 16S rRNA gene amplicon sequencing data and associated metadata from two different studies referred to here as dataset S1 (Wu et al., 2016) and dataset S2 (Beghini et al., 2019) (see the *Datasets* in the METHODS section for more details). Filtering samples for quality-controlled metadata, de-noising of sequencing reads, and sequencing depth filtering resulted in a total of 1,245 samples (*N* = 1,088 from dataset S1 and *N* = 157 from dataset S2). In the whole dataset, class imbalance in smoking habits was large with 512 (44.1%) never smokers, 558 (44.8%) former smokers, and only 175 (14.1%) current smokers. Female samples accounted for 41.5% of the total sample, and the average age (±standard deviation) was 65.2 (±11.0) years. European ancestry of the saliva sample donors was overrepresented (87.3%), as typically encountered in human microbiome data publicly available thus far. The selected characteristics of the two datasets are described in further detail in Table 1.

Microbial taxonomy assignment using the expanded Human Oral Microbiome Database (eHOMD) (v.15.2) (Escapa et al., 2018) as reference (see *Processing of 16S rRNA gene amplicon sequencing data* in the Methods section for more details) and abundance filtering resulted in 200 species from 33 families in dataset S1 and 168 species from 35 families in dataset S2. Both datasets were dominated by a few species that comprised more than 75% of the total microbial composition (21 species in S1 and 15 species in S2). These species belonged to different genera, including *Streptococcus, Rothia, Haemophilus, Prevotella, Veillonella*, and *Actinomyces*. Dataset S1 was dominated by *Streptococcus oralis* (0.26 of total relative abundance), followed by *S. salivarius* (0.09), *Rothia mucilaginosa* (0.06), *S. parasanguinis* (0.05), and *Haemophilus parainfluenzae* (0.05). Dataset S2 was also dominated by *S. oralis* (0.24), followed by *S. parasanguinis* (0.06), *S. salivarius* (0.06), *Prevotella melaninogenica* (0.06), and *R. mucilaginosa* (0.06) (Supplementary Figure 1). These top abundant species were prevalent in both datasets, appearing in more than 87% of individuals. Our observations are consistent with the reported composition of the saliva microbiome (Segata et al., 2012). For downstream analyses, we selected 124 species from 30 families that were common between the two datasets, to ensure that our proposed strategy was generalizable for the prediction of samples from both datasets (Supplementary Table 2). These common species accounted for 86% of the sequencing reads in dataset S1 and 61% in dataset S2.

### Classification of Smoking Habits

The overall saliva microbial communities differed with statistical significance between current and never smokers (ANOSIM *R* = 0.04, *q* = 0.03; PERMANOVA pseudo-*F* = 11.37, *q* = 0.002), and current and former smokers (ANOSIM *R* = 0.04, *q* = 0.03; PERMANOVA pseudo-*F* = 11.91, *q* = 0.002), but not between never and former smokers (ANOSIM *R* = 0, *q* = 0.51; PERMANOVA pseudo-*F* = 0.64, *q* = 0.63). Therefore, we grouped the never and former smokers into a single category of non-current smokers, which when compared with the current smokers showed statistically significant differences in the overall microbial communities (ANOSIM *R* = 0.04, *q* = 0.02; PERMANOVA pseudo-*F* = 13.26, *q* = 0.001). Based on these results, we used two classes of non-current and current smokers in all downstream analyses.

### Validation of Data Types and Machine Learning Models for Smoking Habit Prediction

**Figure 2:**
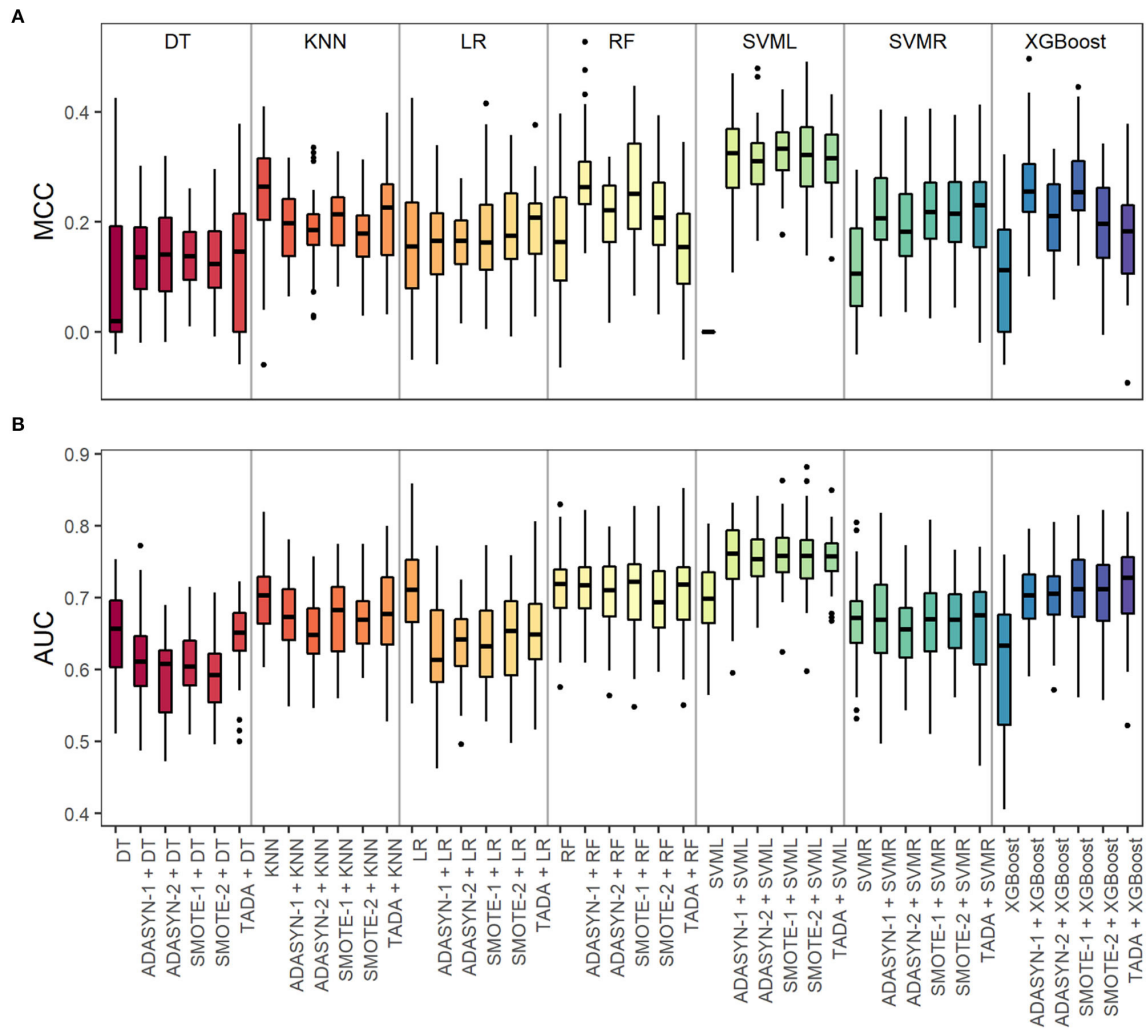
Validation of data types with machine learning (ML) methods for microbiome-based prediction of smoking habits based on the S1 and S2 datasets together. For each ML method, we evaluated six types of input data: baseline non-augmented and five augmented datasets based on different methods (ADASYN-1, ADASYN-2, SMOTE-1, SMOTE-2, and TADA). **(A)** Matthews correlation coefficient (MCC) and **(B)** area under the receiver operating characteristic curve (AUC) values from the 5-fold nested crossed-validation were repeated for 10 times (5 ^*^ 10). For MCC, +1 represents a perfect prediction, 0 random prediction, and −1 perfect inverse prediction. For AUC, 1 indicates perfectly accurate prediction and 0.5 indicates random prediction. ML method abbreviations: DT, decision trees; KNN, k-nearest neighbors; LR, logistic regression; RF, random forest; SVML, support vector machine with linear kernel; SVMR, support vector machine with radial kernel; XGBoost, extreme gradient boosting.

A step-by-step overview of our analytical setup can be found in Figure 1. For each input data type (*d* = 6), including augmented data and baseline non-augmented data, and each ML model (*m* = 7), the resulting classifiers' performance metrics are expressed as Matthews correlation coefficient (MCC) and area under the receiver operating characteristic curve (AUC), which are summarized in Figure 2 and Supplementary Table 3. Overall, data augmentation techniques combined with ML methods outperformed baseline methods based on the MCC values, except for the KNN method. Briefly, the MCC values resulting from the baseline non-augmented methods increased on average when applying data augmentation techniques with percentages of increase as follows: XGBoost (99.8%), SVMR (92.7%), DT (48.9%), RF (30.6%), and LR (8.8%). The highest increase was observed with SVML where the baseline non-augmented method resulted in random prediction (MCC equal or close to zero), which was highly improved with data augmentation techniques (MCC values 0.31–0.33). Notably, the AUC baseline values did not change so drastically when applying data augmentation techniques [percentage increase or decrease (–)]: XGBoost (15.8%), SVML (8%), SVMR (null increase/decrease), RF (−1.0%), KNN (−4.6%), DT (−6.1%), and LR (−10.4%).

The SVML method performed the best in predicting smoking habits from microbiome data based on the MCC metric. As the reference metric for comparison purposes, we chose the MCC, since it is independent of data imbalance, which is not the case for the AUC metric. MCC values were significantly higher with each of the five augmented data types compared to non-augmented data (Wilcoxon test, BH-adjusted *p* = 9.93E−20) (Supplementary Table 4). However, there were no statistically significant differences in the MCC metric between the augmented data pair comparisons (Wilcoxon test, BH-adjusted *p-*values between *p* = 0.392 and *p* = 0.882) (Supplementary Table 4). From these results, we concluded that SVML with augmented data performed better than with imbalanced non-augmented data.

For the training (Figure 1I) and the validation in the holdout test set (Figure 1J) of the final smoking prediction model, we chose SVML combined with TADA. We based our decision on the following: (i) the SVML method performed the best in predicting smoking habits from microbiome data based on the MCC metric (no statistical difference), and (ii) we selected TADA as the preferred data augmentation technique since it takes into account the phylogenetic relationship between the microbial taxa. The average model performance (standard deviation) metrics were MCC of 0.32 (0.07) and AUC of 0.74 (0.05) in the training set, and MCC of 0.36 (0.06) and AUC of 0.81 (0.04) in the holdout test set.

## Discussion

In this study, coming with the available 16S rRNA gene amplicon microbiome sequencing data, we deal with the common issue of data imbalance in human microbiome binary classification, with the aim of unlocking the prediction of human host's traits from saliva microbiome. As a data source, we focused on studies targeting the saliva microbiome and did not use data from studies targeting other niches in the oral cavity due to known diverse microbial assemblies on different oral sites (Aas et al., 2005; Zaura et al., 2009; Segata et al., 2012). We selected publicly available saliva microbiome data from two studies that might differ in their experimental setup (Supplementary Table 1) but both have large sample sizes, while discarding other studies with very small sample sizes that could be a source of variation rather than useful information for the prediction. The lack of widespread consensus on microbiome analysis methods, together with the variation introduced at each step of the microbiome pipeline, constitutes hurdles for cross-study applications. This lack can sometimes outweigh the factor(s) of interest and limit the statistical power and generalization of the application (Brooks et al., 2015; Sinha et al., 2015, 2017; Wang and LêCao, 2019). Though we could not control for any potential variation introduced during the experimental analysis, we aimed to apply the same or the most similar bioinformatics analysis to the raw sequencing data to avoid study-specific computational variation, from which quality control choices are amongst the largest sources of variation (Sinha et al., 2015, 2017). Moreover, we only selected the species common between the datasets from the two studies for downstream analyses. On the one hand, we are aware that this might have reduced the power of our prediction analysis by discarding informative species in each of the two datasets separately. On the other hand, this procedure ensured that the approach is suitable for the prediction of samples from both datasets.

Our observations in the overall microbial composition of saliva were in agreement with the two original studies (Wu et al., 2016; Beghini et al., 2019), where microbiome variation did not significantly differ between never and former smokers (ANOSIM *R* = 0, *q* = 0.51; PERMANOVA pseudo-*F* = 0.64, *q* = 0.63), but significantly differed between never and current (ANOSIM *R* = 0.04, *q* = 0.03; PERMANOVA pseudo-*F* = 11.37, *q* = 0.002), and between former and current smokers (ANOSIM *R* = 0.04, *q* = 0.03; PERMANOVA pseudo-*F* = 11.91, *q* = 0.002). One of the two studies (Wu et al., 2016) also reported significant differences between current smokers and non-current smokers (combined never and former) as we observed in this study (ANOSIM *R* = 0.04, *q* = 0.02; PERMANOVA pseudo-*F* = 13.26, *q* = 0.001).

The available saliva microbiome dataset presents the problem of data imbalance, which is commonly encountered in microbiome datasets and in many other real-life applications, with a ratio of about 1:6 between the minority class of current smokers and the majority class of non-smokers (Table 1). Using class-imbalanced data in prediction modeling can lead to spurious high accuracy based on the correct classification of most of the samples from the majority class at the cost of misclassifying many or even most of the samples from the minority class (Japkowicz and Stephen, 2002; Abd Elrahman and Abraham, 2013; Ali et al., 2013; Thabtah et al., 2020). Regarding our study purpose, this would translate in the classifier's inability to correctly predict the positive observations for current smoking habits (minority class). This was seen in the baseline non-augmented data with the SVML method (Figure 2 and Supplementary Table 3), where we obtained a low MCC of zero but a medium AUC of 0.7. Besides needing to address the class imbalance, this also highlights the necessity of not relying only on a single prediction accuracy score for model validation when dealing with imbalanced data (Chicco and Jurman, 2020).

The MCC performance metric allowed us for fair comparisons of the validated ML methods for both non-augmented and augmented data, since MCC is independent of data imbalance (Boughorbel et al., 2017; Ballabio et al., 2018). For the great majority of the ML methods, augmented data resulted in higher MCC scores compared to imbalanced non-augmented data, thus facilitating improved classification performance. This demonstrates that microbiome-based classification problems can benefit from data augmentation techniques, in line with previous suggestions (Knights et al., 2011). In our dataset, the combined over- and under-sampling approaches generally performed slightly better (though not statistically significantly) than the over-sampling approach alone (Supplementary Tables 2, 3).

The variation in the performance metric values for each input data type and ML method (Figure 2) highlights the variation introduced in the optimization and validation procedures (Figure 1). This underlines the necessity for an nCV approach for overall model validation and selection that is independent of the different data partitions (Cawley and Talbot, 2010; Krstajic et al., 2014). We avoided over-fitting in model validation and classifier selection as demonstrated by the very similar performance metrics between the final training (MCC: 0.32 ± 0.07, AUC: 0.74 ± 0.05) and test (MCC: 0.36 ± 0.06, AUC: 0.81 ± 0.04) datasets, which were very similar to those of the folds in the nCV (MCC: 0.31 ± 0.06, AUC: 0.75 ± 0.05). As it has been suggested before (Topçuoglu et al., 2020), with our strategy, we report the variation in the predictive performance on the different folds of nCV, as well as on both the final training and test sets, which unfortunately is not a very common practice in microbiome-based trait prediction.

With the best data augmentation and ML approach chosen, we predicted individuals' smoking habits from saliva 16S rRNA gene microbiome data in the final holdout test set with MCC of 0.36 and AUC of 0.81. Previously, Sato et al. (2020) predicted smoking habits from class-imbalanced tongue metagenomics data (*N* = 234 never, *N* = 52 current smokers) using an RF approach and conventional non-nested k-fold CV and obtained an AUC of 0.75 from the test set. This prediction was improved to AUC = 0.80–0.93 when using single-nucleotide variants of single species as input data instead of relative abundances of all species. More recently, Carrieri et al. (2021) predicted smoking habits from leg skin 16S rRNA gene amplicon sequencing data based on a less class-imbalanced but very small dataset (*N* = 43 never, *N* = 19 current smokers) using the XGboost method and conventional non-nested k-fold CV and reported the F1 performance metric in the CV folds (F1 = 0.72 ± 0.12), training set (F1 = 0.98), and test set (F1 = 0.85). The noted differences in the F1 scores might be an indication of introduced variation by the different data partitions and bias toward model selection, which can be overcome using an nCV approach as proposed by us and others. Notably, the methods applied in both of these previous studies did not take the class imbalance problem in the used data into account. Therefore, and because of the small sample size in one of these studies at least, the previously reported prediction accuracies are not expected to be reliable, in contrast to the results from our study.

However, in our dataset, we acknowledge some metadata-related characteristics that might limit the prediction of microbiome-based smoking habits, even when the data imbalance issue was accounted for by our approach. Precise phenotype descriptions were available in only one of the two studies (Supplementary Table 1), which is a commonly encountered problem in cross-study applications and can limit the interpretation of results (Huttenhower et al., 2014). Also, the dataset is overrepresented by the age range of 50–79 years old and European ancestry of the sample donors (Table 1), which might result in different prediction performances in other age groups (Lira-Junior et al., 2018; Liu et al., 2020) and ethnicities (Mason et al., 2013; Yang et al., 2019b). To add, one limitation of the data augmentation techniques is that synthesized metadata associated with the synthetically produced data is not reliable. This limits the possibility of statistically adjusting for covariates (i.e., age, sex, and ethnicity) in the ML methods, which can ultimately improve the prediction performance. Hence, the ideal scenario would be to start from a sample that is a good representation of the general population, though this is challenging in real-life applications.

To conclude, by testing different data augmentation techniques and ML methods on class-imbalanced microbiome data, we established a best-practice approach for reliable prediction of individuals' smoking habits from the saliva microbiome that takes the underlying data imbalance into account. We found that combining data augmentation with ML generally outperformed baseline methods in our dataset for our purpose, as other researchers have also suggested before (Knights et al., 2011). The prediction accuracies, expressed as MCC of 0.36 and AUC of 0.81, we achieved for our best model in the final test set implies that predicting human smoking habits from microbiome data needs further improvement before it can be considered for practical applications.

## Data Availability Statement

The original contributions presented in the study are included in the article/Supplementary Material, further inquiries can be directed to the corresponding author/s. The source code and data used for the analyses performed in this study can be accessed at https://github.com/dmontielg/smokingmicrobiome.

## Author Contributions

CD and AV conceptualized the idea. CD, DM, and AV designed the study with contributions by MK. DM implemented and performed the data analysis. CD contributed to initial data collection and curation, performed some of the statistical analyses, and prepared the display items. MK provided resources. CD, AV, and MK wrote the manuscript with contributions by DM. All authors approved the final manuscript.

## Funding

The work of all authors was supported by Erasmus MC University Medical Center Rotterdam. This research received no specific grant from any funding agency in the public, commercial, or not-for-profit sectors.

## Conflict of Interest

The authors declare that the research was conducted in the absence of any commercial or financial relationships that could be construed as a potential conflict of interest.

## Publisher's Note

All claims expressed in this article are solely those of the authors and do not necessarily represent those of their affiliated organizations, or those of the publisher, the editors and the reviewers. Any product that may be evaluated in this article, or claim that may be made by its manufacturer, is not guaranteed or endorsed by the publisher.
