# Supplementary material for: Prediction of Smoking Habits From Class-Imbalanced Saliva Microbiome Data Using Data Augmentation and Machine Learning
Source: Front Microbiol. 2022 Jul 19;13:886201. doi: 10.3389/fmicb.2022.886201 (PMC9343866; doi:10.3389/fmicb.2022.886201)
Supplement: Supplementary file 1 [file Data_Sheet_1.pdf]

Supplementary Material

1 Supplementary Figure and Tables

1.1 Supplementary Figure

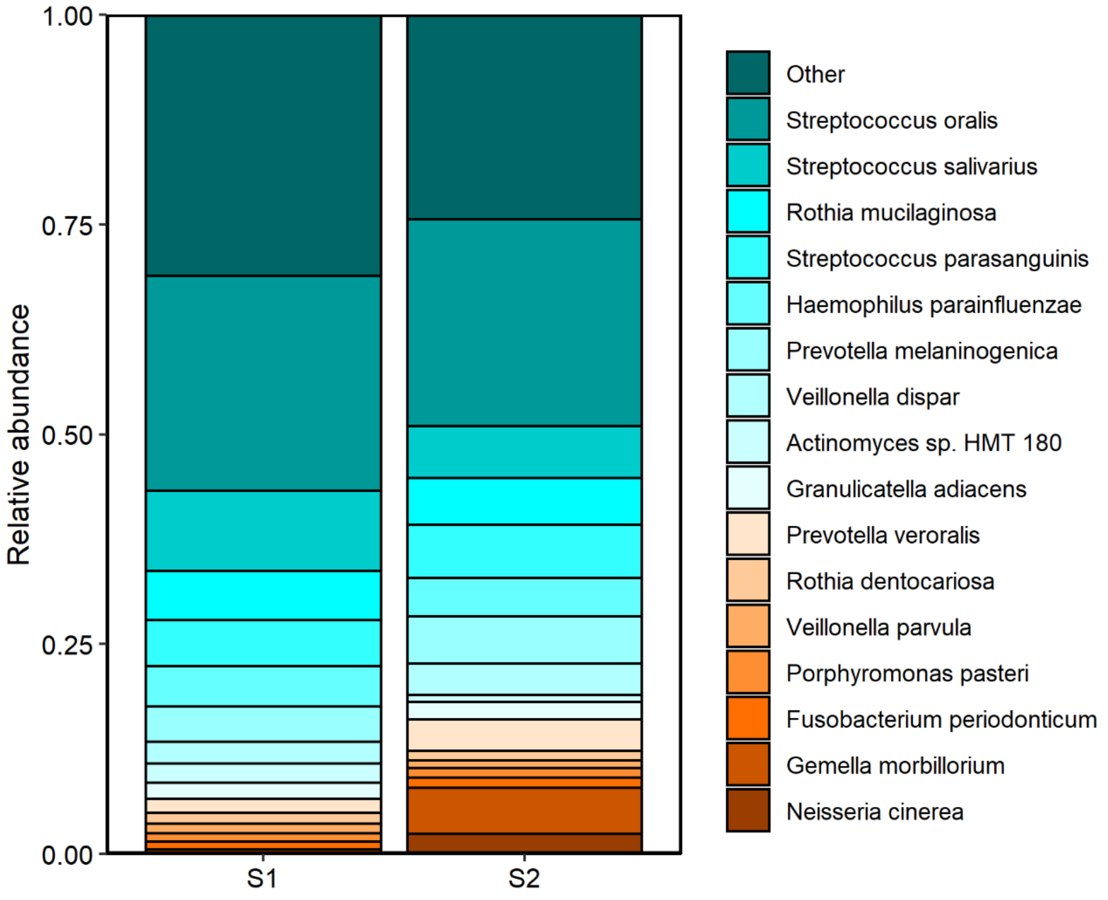

Supplementary Figure 1. Overview of the microbial compositions expressed as the relative abundance of the two analysed datasets.

1.2 Supplementary Tables

Supplementary Table 1. Experimental characteristics of the two analysed studies.

**Study ID**	**S1**	**S2**
**EMBL-EBI Accession Number**	PRJNA434300 (ACS CPS-II cohort) PRJNA434312 (NCI PLCO cohort)	PRJNA484874
**Current smokers definition**	NA	>100 lifetime smoked cigarettes, smoked a cigarette in the last 5 days and did not use any alternative tobacco product in the last 5 days
**Never smokers definition**	NA	<100 lifetime smoked cigarettes, no usage of any tobacco product in the last 5 days and serum cotinine levels less than 0.05 ng/mg
**Former smokers definition**	NA	>100 lifetime smoked cigarettes, currently not smoking, no usage of any tobacco product in the last 5 days and serum cotinine levels less than 0.05 ng/mL
**DNA Isolation Kit**	Mo Bio PowerSoil DNA Isolation Kit (Mo Bio Laboratories)	QIAamp DNA Mini Kit (QIAGEN)
**16S rRNA gene region(s)**	V3-V4	V4
**Sequencing Platform**	454 Roche FLX Titanium	Illumina MiSeq
**Sequencing reads**	Single-end	2x300 paired-end

Supplementary Table 2. List of the 124 species from 30 families that were common between the two analysed datasets.

**Phylum**	**Class**	**Order**	**Family**	**Genus**	**Species**
Absconditabacteria (SR1)	Absconditabacteria (SR1) [C-1]	Absconditabacteria (SR1) [O-1]	Absconditabacteria (SR1) [F-1]	Absconditabacteria (SR1) [G-1]	bacterium HMT 875
Absconditabacteria (SR1)	Absconditabacteria (SR1) [C-1]	Absconditabacteria (SR1) [O-1]	Absconditabacteria (SR1) [F-1]	Absconditabacteria (SR1) [G-1]	bacterium HMT 345
Actinobacteria	Actinobacteria	Actinomycetales	Actinomycetaceae	Actinomyces	sp. HMT 180
Actinobacteria	Actinobacteria	Actinomycetales	Actinomycetaceae	Actinomyces	sp. HMT 169
Actinobacteria	Actinobacteria	Actinomycetales	Actinomycetaceae	Actinomyces	graevenitzii
Actinobacteria	Actinobacteria	Actinomycetales	Actinomycetaceae	Actinomyces	lingnae
Actinobacteria	Actinobacteria	Bifidobacteriales	Bifidobacteriaceae	Alloscardovia	omnicolens
Actinobacteria	Actinobacteria	Bifidobacteriales	Bifidobacteriaceae	Bifidobacterium	dentium
Actinobacteria	Actinobacteria	Bifidobacteriales	Bifidobacteriaceae	Scardovia	wiggsiae
Actinobacteria	Actinobacteria	Bifidobacteriales	Bifidobacteriaceae	Parascardovia	denticolens
Actinobacteria	Coriobacteriia	Coriobacteriales	Coriobacteriaceae	Atopobium	parvulum
Actinobacteria	Coriobacteriia	Coriobacteriales	Coriobacteriaceae	Atopobium	rimae
Actinobacteria	Coriobacteriia	Coriobacteriales	Coriobacteriaceae	Atopobium	NA
Actinobacteria	Actinobacteria	Corynebacteriales	Corynebacteriaceae	Corynebacterium	durum
Actinobacteria	Actinobacteria	Corynebacteriales	Corynebacteriaceae	Corynebacterium	matruchotii
Actinobacteria	Actinobacteria	Actinomycetales	Micrococcaceae	Rothia	mucilaginosa
Actinobacteria	Actinobacteria	Actinomycetales	Micrococcaceae	Rothia	aeria
Actinobacteria	Actinobacteria	Actinomycetales	Micrococcaceae	Rothia	dentocariosa
Bacteroidetes	Bacteroidia	Bacteroidales	Bacteroidales [F-2]	Bacteroidales [G-2]	bacterium HMT 274
Bacteroidetes	Flavobacteriia	Flavobacteriales	Flavobacteriaceae	Capnocytophaga	leadbetteri
Bacteroidetes	Flavobacteriia	Flavobacteriales	Flavobacteriaceae	Capnocytophaga	granulosa
Bacteroidetes	Flavobacteriia	Flavobacteriales	Flavobacteriaceae	Bergeyella	sp. HMT 206
Bacteroidetes	Flavobacteriia	Flavobacteriales	Flavobacteriaceae	Bergeyella	sp. HMT 322
Bacteroidetes	Bacteroidia	Bacteroidales	Porphyromonadaceae	Porphyromonas	pasteri
Bacteroidetes	Bacteroidia	Bacteroidales	Porphyromonadaceae	Porphyromonas	endodontalis

Bacteroidetes	Bacteroidia	Bacteroidales	Porphyromonadaceae	Porphyromonas	gingivalis
Bacteroidetes	Bacteroidia	Bacteroidales	Porphyromonadaceae	Tannerella	forsythia
Bacteroidetes	Bacteroidia	Bacteroidales	Porphyromonadaceae	Porphyromonas	sp. HMT 930
Bacteroidetes	Bacteroidia	Bacteroidales	Prevotellaceae	Prevotella	nanceiensis
Bacteroidetes	Bacteroidia	Bacteroidales	Prevotellaceae	Prevotella	intermedia
Bacteroidetes	Bacteroidia	Bacteroidales	Prevotellaceae	Alloprevotella	sp. HMT 308
Bacteroidetes	Bacteroidia	Bacteroidales	Prevotellaceae	Prevotella	melaninogenica
Bacteroidetes	Bacteroidia	Bacteroidales	Prevotellaceae	Prevotella	pallens
Bacteroidetes	Bacteroidia	Bacteroidales	Prevotellaceae	Alloprevotella	tannerae
Bacteroidetes	Bacteroidia	Bacteroidales	Prevotellaceae	Prevotella	veroralis
Bacteroidetes	Bacteroidia	Bacteroidales	Prevotellaceae	Prevotella	salivae
Bacteroidetes	Bacteroidia	Bacteroidales	Prevotellaceae	Prevotella	sp. HMT 313
Bacteroidetes	Bacteroidia	Bacteroidales	Prevotellaceae	Prevotella	histicola
Bacteroidetes	Bacteroidia	Bacteroidales	Prevotellaceae	Alloprevotella	sp. HMT 473
Bacteroidetes	Bacteroidia	Bacteroidales	Prevotellaceae	Prevotella	nigrescens
Bacteroidetes	Bacteroidia	Bacteroidales	Prevotellaceae	Prevotella	denticola
Bacteroidetes	Bacteroidia	Bacteroidales	Prevotellaceae	Prevotella	oris
Bacteroidetes	Bacteroidia	Bacteroidales	Prevotellaceae	Alloprevotella	sp. HMT 914
Bacteroidetes	Bacteroidia	Bacteroidales	Prevotellaceae	Prevotella	NA
Bacteroidetes	Bacteroidia	Bacteroidales	Prevotellaceae	Prevotella	aurantiaca
Bacteroidetes	Bacteroidia	Bacteroidales	Prevotellaceae	Alloprevotella	rava
Bacteroidetes	Bacteroidia	Bacteroidales	Prevotellaceae	Prevotella	pleuritidis
Bacteroidetes	Bacteroidia	Bacteroidales	Prevotellaceae	Prevotella	sp. HMT 317
Bacteroidetes	Bacteroidia	Bacteroidales	Prevotellaceae	Prevotella	oulorum
Bacteroidetes	Bacteroidia	Bacteroidales	Prevotellaceae	Prevotella	sp. HMT 305
Bacteroidetes	Bacteroidia	Bacteroidales	Prevotellaceae	Prevotella	sp. HMT 396
Firmicutes	Bacilli	Lactobacillales	Aerococcaceae	Abiotrophia	defectiva
Firmicutes	Bacilli	Lactobacillales	Carnobacteriaceae	Granulicatella	adiacens
Firmicutes	Bacilli	Lactobacillales	Carnobacteriaceae	Granulicatella	elegans

Firmicutes	Bacilli	Bacillales	Gemellaceae	Gemella	morbillorum
Firmicutes	Clostridia	Clostridiales	Lachnospiraceae [XIV]	Lachnoanaerobaculum	umeaense
Firmicutes	Clostridia	Clostridiales	Lachnospiraceae [XIV]	Lachnospiraceae [G-2]	bacterium HMT 96
Firmicutes	Clostridia	Clostridiales	Lachnospiraceae [XIV]	Lachnoanaerobaculum	orale
Firmicutes	Clostridia	Clostridiales	Lachnospiraceae [XIV]	Stomatobaculum	sp. HMT 97
Firmicutes	Clostridia	Clostridiales	Lachnospiraceae [XIV]	Stomatobaculum	longum
Firmicutes	Clostridia	Clostridiales	Lachnospiraceae [XIV]	Oribacterium	asaccharolyticum
Firmicutes	Bacilli	Lactobacillales	Lactobacillaceae	Lactobacillus	iners
Firmicutes	Bacilli	Lactobacillales	Lactobacillaceae	Lactobacillus	ultunensis
Firmicutes	Bacilli	Lactobacillales	Lactobacillaceae	Lactobacillus	gasseri
Firmicutes	Bacilli	Lactobacillales	Lactobacillaceae	Lactobacillus	fermentum
Firmicutes	Bacilli	Lactobacillales	Lactobacillaceae	Lactobacillus	salivarius
Firmicutes	Clostridia	Clostridiales	Peptoniphilaceae	Parvimonas	micra
Firmicutes	Clostridia	Clostridiales	Peptostreptococcaceae [XI]	Peptostreptococcaceae [XI][G-1]	sulci
Firmicutes	Clostridia	Clostridiales	Peptostreptococcaceae [XI]	Peptostreptococcus	stomatis
Firmicutes	Clostridia	Clostridiales	Peptostreptococcaceae [XI]	Mogibacterium	diversum
Firmicutes	Clostridia	Clostridiales	Peptostreptococcaceae [XI]	Peptostreptococcaceae [XI][G-9]	brachy
Firmicutes	Clostridia	Clostridiales	Peptostreptococcaceae [XI]	Filifactor	alocis
Firmicutes	Clostridia	Clostridiales	Peptostreptococcaceae [XI]	Mogibacterium	timidum
Firmicutes	Clostridia	Clostridiales	Ruminococcaceae	Ruminococcaceae [G-1]	bacterium HMT 75
Firmicutes	Clostridia	Clostridiales	Ruminococcaceae	Ruminococcaceae [G-2]	bacterium HMT 85
Firmicutes	Negativicutes	Selenomonadales	Selenomonadaceae	Selenomonas	sp. HMT 136
Firmicutes	Negativicutes	Selenomonadales	Selenomonadaceae	Selenomonas	sputigena
Firmicutes	Bacilli	Lactobacillales	Streptococcaceae	Streptococcus	oralis subsp. dentisani clade 58
Firmicutes	Bacilli	Lactobacillales	Streptococcaceae	Streptococcus	salivarius
Firmicutes	Bacilli	Lactobacillales	Streptococcaceae	Streptococcus	mutans
Firmicutes	Bacilli	Lactobacillales	Streptococcaceae	Streptococcus	parasanguinis clade 411
Firmicutes	Bacilli	Lactobacillales	Streptococcaceae	Streptococcus	anginosus
Firmicutes	Bacilli	Lactobacillales	Streptococcaceae	Streptococcus	intermedius

Firmicutes	Bacilli	Lactobacillales	Streptococcaceae	Streptococcus	constellatus
Firmicutes	Bacilli	Lactobacillales	Streptococcaceae	Streptococcus	downei
Firmicutes	Negativicutes	Veillonellales	Veillonellaceae	Veillonella	rogosae
Firmicutes	Negativicutes	Veillonellales	Veillonellaceae	Veillonella	parvula
Firmicutes	Negativicutes	Veillonellales	Veillonellaceae	Veillonella	dispar
Firmicutes	Negativicutes	Veillonellales	Veillonellaceae	Veillonella	sp. HMT 780
Firmicutes	Negativicutes	Veillonellales	Veillonellaceae	Megasphaera	micronuciformis
Firmicutes	Negativicutes	Veillonellales	Veillonellaceae	Veillonella	sp. HMT 917
Firmicutes	Negativicutes	Veillonellales	Veillonellaceae	Dialister	invisus
Firmicutes	Negativicutes	Veillonellales	Veillonellaceae	Dialister	pneumosintes
Fusobacteria	Fusobacteriia	Fusobacteriales	Fusobacteriaceae	Fusobacterium	sp. HMT 203
Fusobacteria	Fusobacteriia	Fusobacteriales	Fusobacteriaceae	Fusobacterium	periodonticum
Fusobacteria	Fusobacteriia	Fusobacteriales	Fusobacteriaceae	Fusobacterium	nucleatum subsp. vincentii
Fusobacteria	Fusobacteriia	Fusobacteriales	Fusobacteriaceae	Fusobacterium	NA
Fusobacteria	Fusobacteriia	Fusobacteriales	Leptotrichiaceae	Leptotrichia	sp. HMT 417
Fusobacteria	Fusobacteriia	Fusobacteriales	Leptotrichiaceae	Leptotrichia	sp. HMT 215
Fusobacteria	Fusobacteriia	Fusobacteriales	Leptotrichiaceae	Leptotrichia	hongkongensis
Fusobacteria	Fusobacteriia	Fusobacteriales	Leptotrichiaceae	Leptotrichia	sp. HMT 221
Fusobacteria	Fusobacteriia	Fusobacteriales	Leptotrichiaceae	Leptotrichia	wadei
Fusobacteria	Fusobacteriia	Fusobacteriales	Leptotrichiaceae	Leptotrichia	sp. HMT 218
Fusobacteria	Fusobacteriia	Fusobacteriales	Leptotrichiaceae	Leptotrichia	shahii
Proteobacteria	Betaproteobacteria	Burkholderiales	Burkholderiaceae	Lautropia	mirabilis
Proteobacteria	Epsilonproteobacteria	Campylobacterales	Campylobacteraceae	Campylobacter	concisus
Proteobacteria	Epsilonproteobacteria	Campylobacterales	Campylobacteraceae	Campylobacter	gracilis
Proteobacteria	Epsilonproteobacteria	Campylobacterales	Campylobacteraceae	Campylobacter	rectus
Proteobacteria	Epsilonproteobacteria	Campylobacterales	Campylobacteraceae	Campylobacter	sp. HMT 44
Proteobacteria	Gammaproteobacteria	Cardiobacteriales	Cardiobacteriaceae	Cardiobacterium	hominis
Proteobacteria	Betaproteobacteria	Neisseriales	Neisseriaceae	Neisseria	NA
Proteobacteria	Betaproteobacteria	Neisseriales	Neisseriaceae	Neisseria	cinerea

Proteobacteria	Betaproteobacteria	Neisseriales	Neisseriaceae	Neisseria	oralis
Proteobacteria	Betaproteobacteria	Neisseriales	Neisseriaceae	Neisseria	flava
Proteobacteria	Betaproteobacteria	Neisseriales	Neisseriaceae	Kingella	oralis
Proteobacteria	Betaproteobacteria	Neisseriales	Neisseriaceae	Kingella	sp. HMT 12
Proteobacteria	Gammaproteobacteria	Pasteurellales	Pasteurellaceae	Haemophilus	parainfluenzae
Proteobacteria	Gammaproteobacteria	Pasteurellales	Pasteurellaceae	Aggregatibacter	segnis
Proteobacteria	Gammaproteobacteria	Pasteurellales	Pasteurellaceae	Haemophilus	paraphrohaemolyticus
Proteobacteria	Gammaproteobacteria	Pasteurellales	Pasteurellaceae	Haemophilus	NA
Proteobacteria	Gammaproteobacteria	Pseudomonadales	Pseudomonadaceae	Pseudomonas	fluorescens
Saccharibacteria (TM7)	Saccharibacteria (TM7) [C-1]	Saccharibacteria (TM7) [O-1]	Saccharibacteria (TM7) [F-1]	Saccharibacteria (TM7) [G-1]	bacterium HMT 352
Saccharibacteria (TM7)	Saccharibacteria (TM7) [C-1]	Saccharibacteria (TM7) [O-1]	Saccharibacteria (TM7) [F-1]	Saccharibacteria (TM7) [G-1]	bacterium HMT 346
Saccharibacteria (TM7)	Saccharibacteria (TM7) [C-1]	Saccharibacteria (TM7) [O-1]	Saccharibacteria (TM7) [F-1]	Saccharibacteria (TM7) [G-6]	bacterium HMT 870

Supplementary Table 3. Validation of data types with machine learning (ML) methods for microbiome-based prediction of smoking habits based on the S1 and S2 datasets together. For each ML method, we evaluated six types of input data as described in the study methods section: baseline non-augmented, and five augmented dataset based on different methods ADASYN-1, ADASYN-2, SMOTE-1, SMOTE-2 and TADA. The Mathews correlation coefficient (MCC) and the area under the receiver operating characteristic curve (AUC) values are the average (and standard deviation) of the performance metrics resulting from the five-fold nested cross-validation repeated for ten times (5*10). For MCC, +1 indicates perfect prediction, 0 random prediction and –1 perfect inverse prediction. For AUC, 1 indicates perfectly accurate prediction and 0.5 indicates random prediction. ML method abbreviations: DT, decision trees; KNN, k nearest neighbors; LR, logistic regression; RF, random forest; SVML, support vector machine with linear kernel; SVMR, support vector machine with radial kernel; XGBoost, extreme gradient boosting.

ML method	Data type	MCC [average (sd)]	AUC [average (sd)]
DT	Non-augmented	0.09 (0.12)	0.65 (0.06)
	ADASYN-1	0.13 (0.08)	0.61 (0.06)
	ADASYN-2	0.14 (0.09)	0.59 (0.05)
	SMOTE-1	0.14 (0.06)	0.61 (0.05)
	SMOTE-2	0.13 (0.07)	0.59 (0.05)
	TADA	0.13 (0.13)	0.65 (0.05)
KNN	Non-augmented	0.25 (0.10)	0.70 (0.05)
	ADASYN-1	0.19 (0.06)	0.67 (0.05)
	ADASYN-2	0.18 (0.07)	0.65 (0.05)
	SMOTE-1	0.20 (0.06)	0.67 (0.06)
	SMOTE-2	0.18 (0.06)	0.67 (0.05)
	TADA	0.21 (0.09)	0.68 (0.06)
LR	Non-augmented	0.16 (0.11)	0.71 (0.07)
	ADASYN-1	0.16 (0.09)	0.62 (0.07)
	ADASYN-2	0.16 (0.06)	0.63 (0.05)
	SMOTE-1	0.18 (0.09)	0.64 (0.06)
	SMOTE-2	0.18 (0.08)	0.64 (0.06)
	TADA	0.19 (0.07)	0.65 (0.06)
RF	Non-augmented	0.17 (0.10)	0.71 (0.05)
	ADASYN-1	0.28 (0.08)	0.72 (0.04)

	ADASYN-2	0.21 (0.07)	0.70 (0.05)
	SMOTE-1	0.26 (0.09)	0.71 (0.06)
	SMOTE-2	0.21 (0.08)	0.70 (0.05)
	TADA	0.15 (0.09)	0.71 (0.05)
SVML	Non-augmented	0.00 (0.00)	0.70 (0.06)
	ADASYN-1	0.31 (0.07)	0.75 (0.05)
	ADASYN-2	0.31 (0.06)	0.76 (0.04)
	SMOTE-1	0.33 (0.06)	0.76 (0.04)
	SMOTE-2	0.32 (0.08)	0.76 (0.05)
	TADA	0.31 (0.06)	0.75 (0.05)
SVMR	Non-augmented	0.11 (0.09)	0.67 (0.06)
	ADASYN-1	0.22 (0.09)	0.67 (0.07)
	ADASYN-2	0.19 (0.07)	0.65 (0.05)
	SMOTE-1	0.22 (0.07)	0.67 (0.06)
	SMOTE-2	0.22 (0.08)	0.67 (0.05)
	TADA	0.21 (0.09)	0.66 (0.07)
XGBoost	Non-augmented	0.11 (0.10)	0.61 (0.08)
	ADASYN-1	0.26 (0.07)	0.70 (0.05)
	ADASYN-2	0.21 (0.08)	0.70 (0.04)
	SMOTE-1	0.26 (0.08)	0.71 (0.05)
	SMOTE-2	0.20 (0.08)	0.70 (0.06)
	TADA	0.17 (0.08)	0.72 (0.06)

Supplementary Table 4. Wilcoxon test pair comparisons between the different types of input data with the support vector machine with linear kernel (SVML). Comparisons are based on the Mathews correlation coefficient (MCC) values resulted from the five-fold nested crossed-validation repeated for ten times (5*10). Significant *p* values were determined with a value cut-off of 0.05 after Benjamini-Hochberg (BH) correction.

**Group 1**	**Group 2**	**BH *p* value**
Non-augmented	ADASYN-1	9.93E-20
Non-augmented	ADASYN-2	9.93E-20
Non-augmented	SMOTE-1	9.93E-20
Non-augmented	SMOTE-2	9.93E-20
Non-augmented	TADA	9.93E-20
ADASYN-1	ADASYN-2	0.595
ADASYN-1	SMOTE-1	0.518
ADASYN-1	SMOTE-2	0.682
ADASYN-1	TADA	0.882
ADASYN-2	SMOTE-1	0.124
ADASYN-2	SMOTE-2	0.416
ADASYN-2	TADA	0.682
SMOTE-1	SMOTE-2	0.741
SMOTE-1	TADA	0.392
SMOTE-2	TADA	0.595
